# Distributed Non-Communicating Multi-Robot Collision Avoidance via Map-Based Deep Reinforcement Learning

**DOI:** 10.3390/s20174836

**Published:** 2020-08-27

**Authors:** Guangda Chen, Shunyi Yao, Jun Ma, Lifan Pan, Yu’an Chen, Pei Xu, Jianmin Ji, Xiaoping Chen

**Affiliations:** 1 School of Computer Science and Technology, University of Science and Technology of China, Hefei 230026, China; cgdsss@mail.ustc.edu.cn (G.C.); ustcysy@mail.ustc.edu.cn (S.Y.); lifanpan@mail.ustc.edu.cn (L.P.); an11099@mail.ustc.edu.cn (Y.C.); xpchen@ustc.edu.cn (X.C.); 2 School of Data Science, University of Science and Technology of China, Hefei 230026, China; markjun@mail.ustc.edu.cn (J.M.); xp816@mail.ustc.edu.cn (P.X.)

**Keywords:** multi-robot navigation, distributed collision avoidance, deep reinforcement learning

## Abstract

It is challenging to avoid obstacles safely and efficiently for multiple robots of different shapes in distributed and communication-free scenarios, where robots do not communicate with each other and only sense other robots’ positions and obstacles around them. Most existing multi-robot collision avoidance systems either require communication between robots or require expensive movement data of other robots, like velocities, accelerations and paths. In this paper, we propose a map-based deep reinforcement learning approach for multi-robot collision avoidance in a distributed and communication-free environment. We use the egocentric local grid map of a robot to represent the environmental information around it including its shape and observable appearances of other robots and obstacles, which can be easily generated by using multiple sensors or sensor fusion. Then we apply the distributed proximal policy optimization (DPPO) algorithm to train a convolutional neural network that directly maps three frames of egocentric local grid maps and the robot’s relative local goal positions into low-level robot control commands. Compared to other methods, the map-based approach is more robust to noisy sensor data, does not require robots’ movement data and considers sizes and shapes of related robots, which make it to be more efficient and easier to be deployed to real robots. We first train the neural network in a specified simulator of multiple mobile robots using DPPO, where a multi-stage curriculum learning strategy for multiple scenarios is used to improve the performance. Then we deploy the trained model to real robots to perform collision avoidance in their navigation without tedious parameter tuning. We evaluate the approach with multiple scenarios both in the simulator and on four differential-drive mobile robots in the real world. Both qualitative and quantitative experiments show that our approach is efficient and outperforms existing DRL-based approaches in many indicators. We also conduct ablation studies showing the positive effects of using egocentric grid maps and multi-stage curriculum learning.

## 1. Introduction

With the rapid development of autonomous mobile robots in recent years, more and more attentions have been paid to multi-robot collision avoidance, which is crucial in many applications, such as multi-robot search and rescue [1], multi-robot intelligent warehouse system [2], autonomous navigation through human crowds [3] and autonomous driving [4]. Multi-robot collision avoidance allows each robot to reach its target position from a starting place while avoiding collisions with other robots and obstacles. The dynamic interactions between diverse autonomous robots and the uncertainty in the environment make the problem highly challenging.

Multi-robot collision avoidance methods can generally be classified into two categories: centralized methods and decentralized methods. A centralized method usually provides a center server to determine each robot’s action using an optimization algorithm after collecting all the relevant information, which has been widely applied in many applications, such as task allocation [5], formation control [6] and object transportation [7,8]. These centralized methods assume that the actions of every robot are determined by a central server with knowledge of every robots’ intents, like initial states and goals, and their surrounding environments. Based on this knowledge, the central server would compute collision avoidance actions by planning optimal paths for every robot simultaneously. Note that centralized methods can generally guarantee safety, completeness and approximate optimality. However, these methods are fragile and difficult to scale to systems with a large number of robots, which is mainly due to the following reasons. Firstly, it becomes more and more challenging for centralized control and scheduling when the number of robots increases. Secondly, a reliable synchronized communication is required between the central server and every robot, which is either uneconomical or not feasible for large-scale systems. Thirdly, the centralized system is vulnerable to various failures, like disturbances of the central server, communication between robots or robots’ motors and sensors. Furthermore, these centralized methods are inapplicable when multiple robots are deployed in an unknown and unstructured environment, especially in a human–robot coexisting environment.

Different from the centralized method, a decentralized method allows each robot to perform collision avoidance relying on its local perception of the surrounding environment, which no longer requires a central server. Here, mobile robots would need to cooperate without necessarily having knowledge of other robot’s intents. Decentralized methods can be roughly divided into two groups, i.e., agent-level and sensor-level, on information specified in each robot’s local perception. In particular, an agent-level method takes into account positions and the movement data, like velocities, accelerations and paths, of other robots. A sensor-level method uses the sensor data directly.

Existing work on agent-level decentralized collision avoidance can be further divided into three categories, i.e., reaction-based, trajectory-based and learning-based. In particular, a reaction-based agent-level decentralized method specifies one-step interaction rules for the current geometric configuration. Velocity Obstacles (VO) [9] are widely used to specify such geometric configurations, which require positions and velocities of other robots to induce the VO for the selection of collision-free motion. Optimal reciprocal collision avoidance (ORCA) [10] is one of the most successful VO-based approaches, which guarantees collision-free trajectories except for densely packed conditions. Specifically, ORCA provides a sufficient condition for multiple robots to avoid collisions with each other in a short time horizon, and can easily be scaled to large systems with many robots. ORCA and its variants [11,12] have been widely used in crowd simulation and multi-agent systems. However, these methods use heuristics to construct parametric collision avoidance models, which are tedious to be tuned for satisfactory performance. These methods also require the perfect sensing of surrounding robots’ positions, velocities and shapes, which is difficult to achieve in real-world applications. Extensions in [13,14,15] require communication networks to share robots’ positions and velocities, which hurt the robustness and flexibility of the system. By using a motion caption system as the global localization infrastructure, Zhou et al. [16] only require positions of surrounding robots using their buffered Voronoi cells. ORCA has also been extended to non-holonomic robots. ORCA-DD [17] doubles the effective radius of each robot to ensure collision-free and smooth paths for robots under differential constraints, which has troubles in narrow passages and unstructured environments. NH-ORCA [18,19] enables a differential drive robot to follow the desired speed command within an error, which only slightly increases the effective radius of a robot and outperforms ORCA-DD in collision avoidance.

On the other hand, a trajectory-based agent-level decentralized method explicitly evaluates the future joint states of robots by anticipating other robots’ future trajectories. A subclass of non-cooperative methods [20,21] first estimates predicted paths of other robots, then plans a collision-free path for the ego robot. However, these predicted paths usually mark a large portion of the space to be unsafe in crowded environments, which leads to the freezing robot problem [22]. To overcome this problem, a subclass of cooperative methods [23,24,25,26] was proposed to take into account the interactions between robots. A cooperative trajectory-based method first infers other robots’ intents, computes jointly feasible paths of all nearby robots, then chooses paths with better quality, e.g., shorter time for every robot to reach their corresponding goals. However, it is hard to estimate other robots’ intents and computationally expensive to generate paths for every robot. Furthermore, the difference between the predicted path and the actual path of each robot grows greater when the time increases, which requires a rapid update for the method.

Decentralized collision avoidance approaches discussed above may require extensive computational efforts or a set of assumptions that may not be met in practice. These approaches also involve a lot of parameters that need to be adjusted manually rather than being able to learn automatically from past experience, which makes them harder to handle unpredictable situations. Learning-based approaches try to optimize a parameterized policy for collision avoidance from experiences in various environments. Deep neural networks have been widely applied in the supervised learning paradigm to train a collision avoidance policy that maps sensor input to the robot’s control commands to navigate a robot in environments with static obstacles [27,28]. Giusti et al. [29] apply neural networks to classify the input color images and determines actions that would keep the quadrotor on the trail. To obtain the large number of training samples, some hikers were equipped with three head-mounted cameras and were required to walk on the mountain path to collect the data. Gandhi et al. [30] create one of the largest crash datasets for drones to train unmanned aerial vehicles to fly. Tai et al. [31] propose a hierarchical structure that combines the decision-making process with a convolutional neural network. Pfeiffer et al. [32] propose a model that is capable to learn complex mappings directly from the 2D laser range results to corresponding control commands of a mobile indoor robot. However, these methods require a massive labeled dataset for the training and suffer from the distribution mismatch between the training dataset and the testing environment, which limits their applications in the real world. Some of them are just able to avoid simple static obstacles in empty corridors.

On the other hand, deep reinforcement learning (DRL) approaches have achieved significant success in many challenging tasks, such as Go game [33,34], video games [35,36] and robotics [37,38]. In contrast, a DRL-based method is able to learn from a large number of trials and corresponding feedbacks (rewards). In order to learn sophisticated control strategies through DRL, the robot needs to interact with the training environment for a long time to accumulate experience about the consequences of taking different actions in different states. However, collecting such interactive data in the real world is very expensive, time-consuming and sometimes impossible due to security issues [39]. Inspired by VO-based approaches, Chen et al. [40] provide a DRL-based method to train an agent-level collision avoidance policy, where the network still requires the expensive movement data of the ego robot, its neighbors and moving obstacles as its inputs. In their extension [41], multiple perception tasks, like segmentation, recognition and tracking, are performed on multiple sensors to estimate the movement data of nearby robots and moving obstacles. However, these perception tasks require extensive computational efforts for online utilization. Notice that, though agent-level methods have the disadvantage of requiring a precise and complex front-end perception procedure, they have the advantages of adaptable to various sensors, easy to be trained in simulation environments and easy to be deployed to a real robot.

To alleviate the disadvantage of the agent-level decentralized collision avoidance methods, Long et al. [42] and Fan et al. [43] provide sensor-level decentralized collision avoidance policies that directly map raw sensor data, i.e., 2D laser scan data, to robot’s control commands. Notice that the difference between the laser data in a simulator and the real world is limited. Then these collision avoidance policies can be trained in various simulation environments and can later be deployed to robots in the real world. However, these methods are only restricted to specific sensors, i.e., 2D laser sensors. Moreover, multiple sensors and their fusion are required for mobile robots to navigate autonomously and safely [44]. For instance, a 2D laser sensor has troubles to handle 3D collision avoidance in an office-like environment, where an RGB-D camera, like Kinect, can be helpful.

In this paper, we propose a decentralized map-based DRL approach for multi-robot collision avoidance in a distributed and communication-free environment. Compared to agent-level [41] and sensor-level [43] methods, we use the egocentric local grid map of a robot to represent the environmental information around it including its shape and observable appearances of other robots and obstacles, which can be easily generated by using multiple sensors or sensor fusion. Then we apply the distributed proximal policy optimization (DPPO) algorithm to train a convolutional neural network that directly maps three frames of egocentric local grid maps and the robot’s relative local goal positions into low-level robot control commands. Our map-based method has the advantages of both agent-level and sensor-level methods, the map-based approach is adaptable to various sensors, easy to be trained in simulation environments, more robust to noisy sensor data, does not require robots’ movement data and a precise and complex front-end perception procedure, and considers sizes and shapes of related robots, which make it to be more efficient, robust and easier to be deployed to real robots. We first train the neural network in a specified simulator of multiple mobile robots using DPPO, where a two-stage curriculum learning strategy for two scenarios is used to improve the performance. Then we deploy the trained model to real robots to perform collision avoidance in their navigation without tedious parameter tuning. We evaluate the approach with multiple scenarios both in the simulator and on four differential-drive mobile robots in the real world. Both qualitative and quantitative experiments show that our approach is efficient and outperforms existing DRL-based approaches in many indicators. We also conduct ablation studies showing the positive effects of using egocentric grid maps and multi-stage curriculum learning.

Our main contributions are summarized as follow:

- We propose a map-based DRL multi-robot collision avoidance approach in a communication-free environment, where egocentric local grid maps are used to represent the environmental information around the robot, which can be easily generated by using multiple sensors or sensor fusion.
- We train the collision avoidance policy in multiple simulation environments using DPPO, which can be deployed to real robots without tedious parameter tuning, where the network considers egocentric local grid maps as inputs and directly outputs low-level robot control commands.
- We evaluate our approach with multiple scenarios both in the simulator and on many differential-drive mobile robots in the real world. Both qualitative and quantitative experiments show that our approach is efficient and outperforms existing related approaches in many indicators.
- We conduct ablation studies that specify the positive effects of using egocentric grid maps and multi-stage curriculum learning.

The rest of this paper is organized as follows. The formulation of multi-robot collision avoidance is presented in Section 2. The applied DRL algorithm for multi-robot collision avoidance based on egocentric local grid maps is described in Section 3. Section 4 and Section 5 describe the simulation experimental results and the real-world experiments respectively, followed by conclusions in Section 6.

## 2. Problem Formulation

Multi-robot collision avoidance requires a group of *N* mobile robots with holonomic or non-holonomic constraints to perform corresponding trajectories to their target places while avoiding collisions with each other and with obstacles in their environment.

In particular, at each time step *t*, each robot *i* (0≤i≤N−1) first receives its sensing data $c_{t}^{i}$, the offset orientation angle $\alpha_{t}^{i}$ relative to its starting pose and a local goal position $g_{t}^{i}$ in its coordinate system, which contains robot *i*’s relative position in a path that is generated by its global planner to reach its target. Then the robot chooses an action $a_{t}^{i}$ to move towards its local goal $g_{t}^{i}$. Note that sensing data $c_{t}^{i}$ does not need to have full observation of the environment. Then without assuming perfect sensing, we only need a partial observation here. On the other hand, as discussed in Section 1, we also do not directly apply raw sensor data as inputs of our collision avoidance policy. Instead, we use the egocentric local grid map $M_{t}^{i}$ to represent the environmental information around the robot *i* including its shape $\Omega^{i}$ and the sensing result $c_{t}^{i}$. In specific, we consider

$$M_{t}^{i}=f_{\lambda }\left(\;c_{t}^{i},\;\Omega^{i}\;\right),$$

where $f_{\lambda }$ denotes the grid map generator specified by parameters λ to generate the egocentric local grid map $M_{t}^{i}$ from the robot’s shape $\Omega^{i}$ and the sensing result $c_{t}^{i}$.

Note that intents and movement data of other robots are not required here. However, we can estimate motion information, i.e., velocities, accelerations and paths, of other robots implicitly from consecutive frames of egocentric local grid maps. We can also estimate ego robot’s historical trajectory and velocities, i.e., linear velocities and angular velocities, from consecutive frames of its local goals and offset orientation angles. In particular, we specify the observation of the robot *i* at time *t* as $o_{t}^{i}=\left(\;M_{t}^{i},\;g_{t}^{i},\;\alpha_{t}^{i}\;\right)$. We can use m≥1 consecutive frames of such observation states as inputs of the collision avoidance policy $\pi_{\theta }$. In specific, we consider

$$a_{t}^{i}=\pi_{\theta }\left(\;o_{t-m+1}^{i},\;\ldots ,\;o_{t}^{i}\;\right),$$

where $\pi_{\theta }$ denotes the collision avoidance policy specified by parameters θ to choose an action $a_{t}^{i}$ based on *m* consecutive frames of the observation. In this paper, we denote action $a_{t}^{i}=\left(v_{t}^{i},\;\omega_{t}^{i}\right)$, where $v_{t}^{i}$ is the linear velocity and $\omega_{t}^{i}$ is the angular velocity that the robot *i* needs to perform until the next time step t+1.

In multi-robot collision avoidance, each robot *i* moves from the starting position $p_{0}^{i}$ to the target position $p_{g}^{i}$, while avoiding collisions with each other and with obstacles $B_{k}$(1≤k≤M), i.e., observable appearances of the obstacles, in the environment. In addition, $B_{k}$ can be considered as a set of cells that are occupied by the corresponding obstacle in the gird map and with a slight abuse of notation, $\Omega^{i}\left(p_{t}^{i}\right)$ denotes a set of cells that are occupied by the robot *i* at the position $p_{t}^{i}$ at time *t* with the shape $\Omega^{i}$. We intend to minimize the expectation of the arrival time $t_{g}^{i}$ for every robot 0≤i≤N−1 under the constraint that no collision occurs with other robots and obstacles. In specific,

$$\begin{array}{c} \underset{\theta }{\arg\min}\;E\left[\;\sum_{i=0}^{N-1}t_{g}^{i}|\pi_{\theta }\right],\\ \;s.t.\;\mathrm{for}\;\mathrm{each}\;0\leq i\leq N-1,\;0\leq j\leq N-1,\;i\neq j,\;\mathrm{and}\;1\leq k\leq M,\\ p_{t+1}^{i}=p_{t}^{i}+\Delta t\cdot \pi_{\theta }\left(o_{t-m+1}^{i},\ldots ,o_{t}^{i}\right),\;p_{t_{g}^{i}}^{i}=p_{g}^{i},\;\Omega^{i}\left(p_{t}^{i}\right)\cap \Omega^{j}\left(p_{t}^{j}\right)=\emptyset ,\;\mathrm{and}\;\Omega^{i}\left(p_{t}^{i}\right)\cap B_{k}=\emptyset . \end{array}$$

Notice that the second line of conditions requires each robot to reach its target without collision. In the following, we introduce our approach towards the intent.

## 3. Approach

We begin this section by describing the key ingredients of the distributed proximal policy optimization (DPPO) reinforcement learning algorithm for multiple robots. Then, we describe the details on the network architecture and the training process for the multi-robot collision avoidance policy. Finally, we elaborate on the training protocols and scenarios used to optimize the policy.

### 3.1. Reinforcement Learning Components

As discussed in Section 2, our formulation of the multi-robot collision avoidance problem can be considered as a partially observable decision-making problem under uncertainty, which can be specified as a Partially Observable Markov Decision Process (POMDP) problem [45] and solved using reinforcement learning algorithms. In specific, a POMDP problem consists of 6 tuples 〈S,A,P,R,Ω,O〉, where S is the state space, A is the action space, P is the transition function that describes the probability of transiting to the next state, R is the reward function that illustrates the immediate state-action reward signal, Ω is the observation space with o∈Ω and *O* is the observation probability function that describes the probability of observing o.

In the following, we describe the details of our map-based approach for multi-robot collision avoidance, including the observation space, the action space and the reward function.

#### 3.1.1. Observation Space

As specified in Section 2, the observation $o_{t}^{i}$ consists of the generated grid map $M_{t}^{i}$, the relative local goal position $g_{t}^{i}$ and the offset orientation angle $\alpha_{t}^{i}$ for the corresponding robot *i*. We use the latest three frames of observations ${\vec{o}}_{t}^{i}=\left(o_{t-2}^{i},\;o_{t-1}^{i},\;o_{t}^{i}\right)$ as inputs of the neural network for the collision avoidance policy. In this paper, the egocentric local grid map $M_{t}^{i}$ is generated by a 2D laser scan with 180 degrees horizontal Field of View (FOV), which encodes robot *i*’s shape and observable appearances of nearby obstacles and other robots. Note that egocentric local grid maps are directly constructed from costmaps (http://wiki.ros.org/costmap_2d) in robotics, which can be easily generated by using various sensors or sensor fusion. The relative local goal position $g_{t}^{i}$ is a two-dimensional vector representing a position (x,y) w.r.t. the current position of the robot *i*, which specifies a position in the path that is generated by the global planner to reach robot *i*’s final target in the environment. The angle $\alpha_{t}^{i}$ represents the change of the robot *i*’s orientation relative to its starting pose. Notice that motion information of other robots can be implicitly extracted from consecutive frames of egocentric local grid maps, and the historical trajectory and velocities of ego robot can be implicitly extracted from consecutive frames of its local goals and offset orientation angles. There is a trade-off between the accuracy of the estimation and the efficiency of the policy. In our experiments, satisfactory performance is achieved when three frames of observations are considered as inputs of the network.

**Figure 1:**
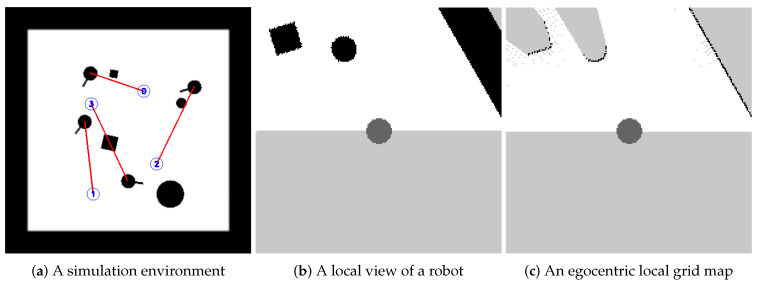
(**a**) A training environment of the simulator, where blue digital circles represent the target positions of robots with the corresponding number, red lines specify the straight paths from the current position to the target for robots and other black pixels represent various obstacles. (**b**) The local view of the environment for the robot 0. (**c**) The egocentric local grid map generated from the local view using a 2D laser sensor.

Egocentric local grid maps are constructed from costmaps, which have been widely applied in robot navigation [46,47] and can be generated from various sensors or sensor fusion with strong noise resistance. We first receive a local costmap w.r.t. the robot *i*, then we can construct the egocentric local grid map $M_{i}^{t}$ by adding the robot’s configuration, i.e., its size and shape, in the costmap. There is a value in each cell of $M_{t}^{i}$ to represent the surrounding environment. In particular, cells of value 0 denote the obstacles and other robots around the robot. Cells of value 255 denote the free space, cells of value 100 denote the undetected place that is unknown for the robot and cells of value 200 denote the place that is occupied by the robot itself. An example of the training environment in the simulator is illustrated in Figure 1a. The generated egocentric local grid map for the robot 0 in this example is shown in Figure 1c.

In this paper, we convert the egocentric local grid map to a gray image, where values of pixels are divided by 255 for normalization, to ease the set up of the network. Without loss of generality, we identity a grid map with its corresponding gray image in the paper.

#### 3.1.2. Action Space

The action space of robots is a set of permissible velocities in continuous space. The action $a_{t}^{i}$ of a differential robot *i* consists of a linear velocity $v_{t}^{i}$ and an angular velocity $\omega_{t}^{i}$, i.e., $a_{t}^{i}=\left(v_{t}^{i},\omega_{t}^{i}\right)$. In this paper, we set $v_{t}^{i}\in \left[0,\;0.6\right]$ (in meters per second) and $\omega_{t}^{i}\in \left[-0.9,\;0.9\right]$ (in radians per second), which can be directly performed by the differential robots used in our experiments. Note that $v_{t}^{i}\geq 0$, i.e., moving backward is not allowed, due to lack of rear sensors.

#### 3.1.3. Reward Function

The goal of the agent is to maximize its cumulative reward in reinforcement learning and the reward function specifies what the agent needs to achieve but not how to achieve it. In our long-distance multi-robot collision avoidance task, the objective is to minimize the mean arrival time for each robot arriving its local goal under the collision-free constraint, where the reward signal is often weak and sparse unless the local goal has been reached or a collision has occurred. To alleviate this problem, we apply the reward shaping technique [48] by adding an extra reward signal $r_{t}^{g}$ to guide the robot to move towards its local goal.

Each robot has the same reward function in our setting. We use $r_{t}$ to denote the reward received by the robot at time *t*. We use the following reward function in this paper,

$$r_{t}=r_{t}^{g}+r_{t}^{a}+r_{t}^{c}+r_{t}^{s}.$$

Note that $r_{t}$ is the sum of four parts, $r_{t}^{g}$, $r_{t}^{a}$, $r_{t}^{c}$ and $r_{t}^{s}$.

In particular, $r_{t}^{g}$ specifies the penalty when the robot goes far of its local goal. We define $r_{t}^{g}$ as:

$$r_{t}^{g}=\eta \left(\;∥p_{t-1}-p_{g}∥-∥p_{t}-p_{g}∥\;\right),$$

where $p_{t}$ is the position of the robot at time *t*, $p_{g}$ is the position of the local goal and η is the hyper-parameter that controls the penalty amount.

$r_{t}^{a}$ denotes the reward when the robot arrives its local goal, i.e., the distance between the robot and its local goal is less than $d_{arr}$, where $r_{\mathrm{arr}}>0$ is the consistent reward.

$$r_{t}^{a}=\left\{\begin{array}{ll} r_{arr} & \mathrm{if}\;∥p_{t}-p_{g}∥<d_{arr},\\ 0 & \mathrm{otherwise}. \end{array}\right.$$

$r_{t}^{c}$ specifies the penalty when the robot encounters a collision. Note that DPPO considers stochastic policies, we add a penalty when the robot gets closer to obstacles or other robots. We define $r_{t}^{c}$ as:

$$r_{t}^{c}=\left\{\begin{array}{ll} r_{\mathrm{col}} & \mathrm{if}\;\mathrm{collision},\\ 0 & \mathrm{otherwise}, \end{array}\right.$$

where $r_{\mathrm{col}}<0$ is the consistent penalty for the collision.

At last, we apply a small negative penalty for each time step, i.e., $r_{t}^{s}<0$, to encourage short paths. In this work, we set $r_{\mathrm{arr}}$ = 500, η = 200, $r_{\mathrm{col}}=-500$ and $r_{t}^{s}=-5$ in the training procedure.

### 3.2. Distributed Proximal Policy Optimization

In the POMDP setting, multi-robot collision avoidance requires a stochastic policy $\pi_{\theta }\left(a_{t}^{i}|{\vec{o}}_{t}^{i}\right)$ for each robot *i*, which specifies the probability of mapping the latest three frames of observations ${\vec{o}}_{t}^{i}$, i.e., $\left(o_{t-2}^{i},o_{t-1}^{i},o_{t}^{i}\right)$, to an action $a_{t}^{i}$ at time *t*. Note that every robot shares the same stochastic policy in our work. Then without loss of generality, we omit the subscript *i* in the following.

In DRL, policy gradient methods optimize a stochastic policy $\pi_{\theta }$ by maximizing the expected return $J(\pi_{\theta })$ using stochastic gradient ascent. In specific,

$$\begin{array}{cc} \nabla_{\theta }J\left(\pi_{\theta }\right) & =\nabla_{\theta }\underset{\tau ∼\pi_{\theta }}{E}\left[R(\tau )\right],\\ \theta_{k+1} & =\theta_{k}+\alpha \nabla_{\theta }J\left(\pi_{\theta_{k}}\right), \end{array}$$

where τ is a trajectory, R(τ) is the finite-horizon discounted return ($R\left(\tau \right)=\sum_{t=0}^{\infty }\gamma^{t}r_{t}$, where γ∈(0,1) is a discount factor), The gradient of policy performance, $\nabla_{\theta }J\left(\pi_{\theta }\right)$, is called the policy gradient and α is the learning rate. By introducing the trajectory probability P(τ|θ) and using mathematical techniques [49], we can further derive the following analytical gradient from the above equation,

$$\begin{array}{cc} \nabla_{\theta }J\left(\pi_{\theta }\right) & =\underset{\tau ∼\pi_{\theta }}{E}\left[\nabla_{\theta }\log P\left(\tau |\theta \right)R\left(\tau \right)\right]\\ \; & =\underset{\tau ∼\pi_{\theta }}{E}\left[\sum_{t=0}^{T}\nabla_{\theta }\log\pi_{\theta }\left(a_{t}|{\vec{o}}_{t}\right)R\left(\tau \right)\right]. \end{array}$$

Generally, we do not need to describe the absolute meaning of an action, but only need to describe how much better it is on average than other actions. In other words, we need to know the advantage of the action. Therefore, we define ${\hat{A}}^{\pi_{\theta }}$ as an estimator of the advantage function for the policy $\pi_{\theta }$, and the above equation is further rewritten as

$$\nabla_{\theta }J\left(\pi_{\theta }\right)=\underset{\tau ∼\pi_{\theta }}{E}\left[\sum_{t=0}^{T}\nabla_{\theta }\log\pi_{\theta }\left(a_{t}|{\vec{o}}_{t}\right){\hat{A}}^{\pi_{\theta }}\left({\vec{o}}_{t},a_{t}\right)\right].$$

While it is appealing to perform multiple steps of optimization on $J(\pi_{\theta })$ using the same trajectory, which is not well-justified and empirically often leads to destructively large policy updates. Proximal Policy Optimization (PPO) [50] addresses this problem by introducing importance sampling and using the clip method to restrict the size of the policy update. In specific, PPO with clip loose updates policies via

(1) $$\theta_{k+1}=\arg\max_{\theta }E_{\vec{o},a∼\pi_{\theta_{k}}}\left[L\left(\vec{o},a,\theta_{k},\theta \right)\right],$$

and *L* is defined as

(2) $$\begin{array}{cc} L\left(\vec{o},a,\theta_{k},\theta \right) & =\min\left(\frac{\pi_{\theta }\left(a|\vec{o}\right)}{\pi_{\theta_{k}}\left(a|\vec{o}\right)}{\hat{A}}^{\pi_{\theta_{k}}}\left(\vec{o},a\right),\;g\left(\epsilon ,{\hat{A}}^{\pi_{\theta_{k}}}\left(\vec{o},a\right)\right)\right),\\ g(\epsilon ,\hat{A}) & =\left\{\begin{array}{cc} \left(1+\epsilon \right)\hat{A} & \hat{A}\geq 0,\\ \left(1-\epsilon \right)\hat{A} & \hat{A}<0, \end{array}\right. \end{array}$$

where ϵ is the clip function ration.

A variance-reduced advantage-function estimator ${\hat{A}}^{\pi_{\theta }}\left({\vec{o}}_{t},a_{t}\right)$ can be constructed using a learned state-value function $V_{\phi }\left({\vec{o}}_{t}\right)$. In particular, we use a truncated version of the generalized advantage estimation (GAE) [51] for a given length-*T* trajectory segment, i.e.,

(3) $${\hat{A}}^{\pi_{\theta }}\left({\vec{o}}_{t},a_{t}\right)=\delta_{t}+\left(\gamma \lambda \right)\delta_{t+1}+\cdots +{\left(\gamma \lambda \right)}^{T-1-t}\delta_{T-1},$$

where $\delta_{t}=r_{t}+\gamma V_{\phi }\left({\vec{o}}_{t+1}\right)-V_{\phi }\left({\vec{o}}_{t}\right)$, $V_{\phi }$ represents the state-value function, the discount factor 0≤γ<1 and the parameter 0≤λ≤1.

In this paper, we apply the distributed Proximal Policy Optimization (DPPO) algorithm to train the stochastic policy for multi-robot collision avoidance. DPPO is extended from PPO by collecting experiences in a distributed setting from a variety of environments where multiple robots share the same policy $\pi_{\theta }$ to take actions interactively. Then these collected experiences are used to update the parameters of the policy $\pi_{\theta }$ and the state-value function $V_{\phi }$.

In the following, we introduce details of our DPPO algorithm, including the training process, the network architecture and the usage of multi-stage curriculum learning.

#### 3.2.1. Training Process

We implement our DPPO algorithm through the centralized learning decentralized execution paradigm, in which homogeneous learning agents sample simultaneously from multiple simulation environments to train the same shared neural network that represents their collision avoidance policy. Algorithm 1 specifies the training process that samples trajectories by executing the policy in parallel and updates the policy with the sampled data. The policy $\pi_{\theta }$ is trained with these collected experiences by all robots simultaneously. Every robot exploits the same policy to generate trajectories in corresponding environments. An episode for robots running in an environment is terminated, when every robot reaches its target or encounters a collision with an obstacle or another robot. Then the environment is also reset. In specific, each robot *i* receives its own inputs ${\vec{o}}_{t}^{i}$, i.e., the latest three frames of observations, at each time step *t* and executes the action $a_{t}^{i}$ generated from the shared policy $\pi_{\theta }$ in multiple environments and then gets a return $r_{t}^{i}$ and a new state observation ${\vec{o}}_{t+1}^{i}$, we store the $\left({\vec{o}}_{t}^{i},a_{t}^{i},r_{t}^{i},V_{t}^{i}\right)$ in the buffer queue (*ll.* 7–11). When the buffer queue is full or the trajectory length reaches the maximum trajectory length limit $T_{m}$ in the episode, we need to set $V_{t+1}^{i}=V_{\phi }\left({\vec{o}}_{t+1}^{i}\right)$ to forcefully cut the trajectory to end the episode (*ll.* 12–13). When the robot reaches the target point ($∥p_{t}^{i}-p_{g}^{i}∥<d_{arr}$), or collides with other obstacles (there exists some 1≤k≤M, s.t., $\Omega^{i}\left(p_{t}^{i}\right)\cap B_{k}\neq \emptyset$), or collides with other robots (there exists some 0≤j≤N−1, s.t., i≠j and $\Omega^{i}\left(p_{t}^{i}\right)\cap \Omega^{j}\left(p_{t}^{j}\right)\neq \emptyset$), we set $V_{t+1}^{i}=0$ to finish the episode (*ll.* 14–15). When the episodes of all robots have finished, we estimate advantages of each step stored in the buffer, and save it to the corresponding position in the buffer, and then reset the simulation environment (*ll.* 18–22).
**Algorithm 1:** Distributed Proximal Policy Optimization
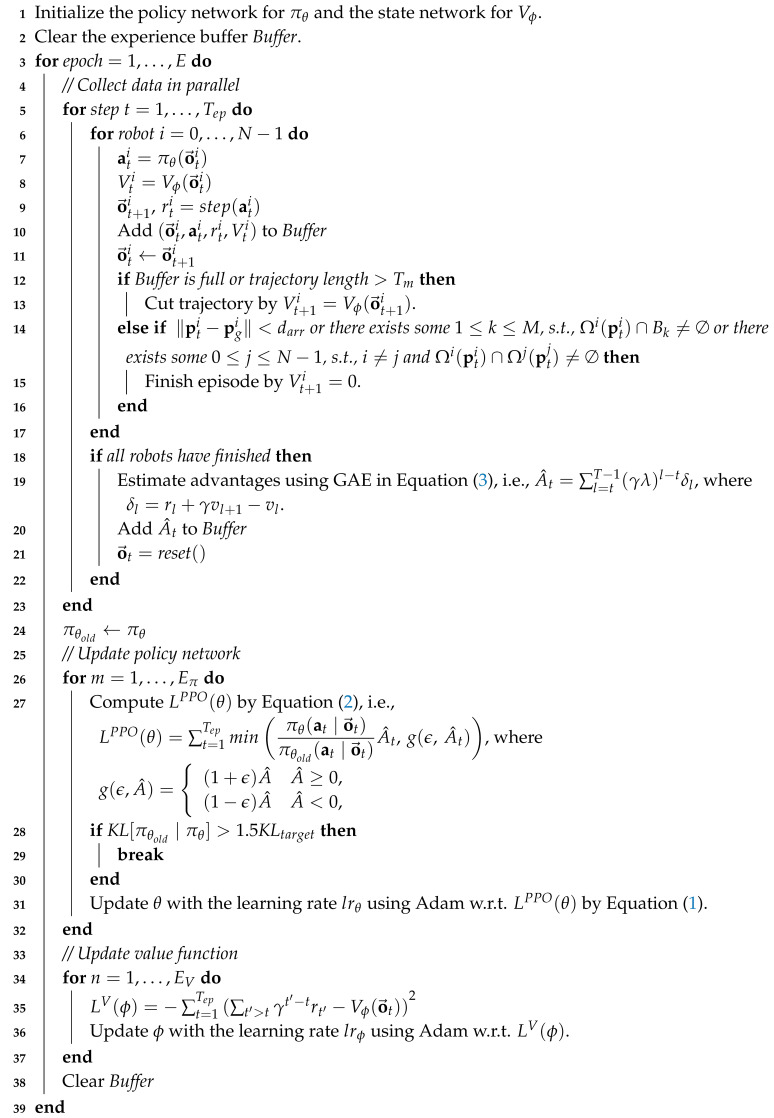



The policy network for the stochastic policy $\pi_{\theta }$ and the value network for the state-value function $V_{\phi }$ are updated when the collected experiences have filled the entire experience buffer. To update the policy $\pi_{\theta }$, the surrogate loss $L^{PPO}\left(\theta \right)$ in Algorithm 1 is constructed from the collected trajectories, which is optimized using the Adam optimizer [52] for $E_{\pi }$ epochs under the Kullback–Leiber (KL) divergence constraint (*ll.* 26–32). To update the state-value function $V_{\phi }$, i.e., the baseline to estimate the advantage function ${\hat{A}}_{t}$, the loss squared-error $L^{V}\left(\phi \right)$ in Algorithm 1 is also constructed from these collected trajectories, which is also optimized using the Adam optimizer for $E_{V}$ epochs (*ll.* 34–37). Finally, we clear the buffer and recollect the experience used for training (*l.* 38). The value network has the same architecture as the policy network, except the last layer is modified to only output the value of ${\vec{o}}_{t}$, i.e., one unit with a linear activation. Note that both the policy network and the value network are updated independently and their parameters are not shared, which due to the fact that using two separate networks often leads to better performance in practice.

The DPPO algorithm in Algorithm 1 can be easily scaled to a multi-robot system with a hundred robots in distributed and communication-free scenarios. We also illustrate such scenarios in our experiments, where each robot is independent to collect its experiences. Note that the decentralized execution not only dramatically reduces the time of sample collection, but also makes the algorithm suitable for training many robots in multiple scenarios.

#### 3.2.2. Network Architecture

**Figure 2:**
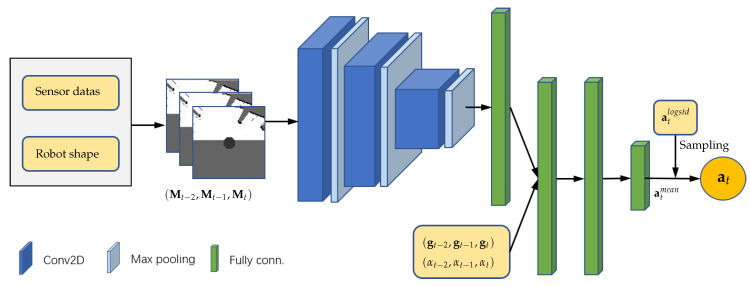
The architecture of the policy network. The input of the network is ${\vec{o}}_{t}$, which consists of three frames of egocentric local grid maps and three frames of local goal positions and offset orientation angles. The network computes the mean of the action, which consists of a linear velocity and an angular velocity. The resulting action is sample from a Gaussian distribution.

The architecture of our policy network for the collision avoidance policy $\pi_{\theta }$ is shown in Figure 2. As introduced in Section 3.1.1, ${\vec{o}}_{t}$ is considered as the input of the network, which consists of three parts, i.e., $\left(M_{t-2},M_{t-1},M_{t}\right)$, three consecutive frames of egocentric local grid maps, $\left(g_{t-2},g_{t-1},g_{t}\right)$, three consecutive frames of relative local goal positions and $\left(\alpha_{t-2},\alpha_{t-1},\alpha_{t}\right)$, three consecutive frames of offset orientation angles.

The network first produces feature maps for grid maps, i.e., $\left(M_{t-2},M_{t-1},M_{t}\right)$, using three convolutional layers $L^{CV}$ and three max pooling layers $L^{MP}$. The output value of the 2D convolutional layer $L^{CV}$ with input size $\left(N,C^{l-1},H^{l-1},W^{l-1}\right)$ and output $\left(N,C^{l},H^{l},W^{l}\right)$ can be precisely described as:

$$x^{l}\left(N_{i},C_{j}^{l}\right)=\mathrm{ReLU}\left(\sum_{k=0}^{C^{l-1}-1}W^{l}\left(C_{j}^{l},k\right)🟉x^{l-1}\left(N_{i},k\right)+b^{l}\left(C_{j}^{l}\right)\right),$$

where ReLU(x)=max(0,x) denotes the rectified linear unit function [53], 🟉 is the valid 2D cross-correlation operator, $x^{l}$ is the output tensor of the network layer *l*, *N* is a batch size, *C* denotes a number of channels, *H* is a height of input planes in pixels and *W* is width in pixels. The output value of the 2D max pooling layer $L^{MP}$ with input size $\left(N,C,H^{l-1},W^{l-1}\right)$, output size $\left(N,C,H^{l},W^{l}\right)$ and kernel size (kH,kW) can be precisely described as:

$$x^{l}\left(N_{i},C_{j},h,w\right)=\overset{kH-1}{\underset{m=0}{max}}\;\overset{kW-1}{\underset{n=0}{max}}\;x^{l-1}\left(N_{i},C_{j},s_{0}\times h+m,s_{1}\times w+n\right),\;\;m,n\in N,$$

where $\left(s_{0},s_{1}\right)$ represents the stride of the window to take a max over, $h\in [0,H^{l}]$ and $w\in [0,W^{l}]$. The first 2D convolutional layer $L_{0}^{CV}$ convolves 64 two-dimensional filters (Conv2D) with kernel 3×3 and stride 1 over the three grid maps, and applies ReLU nonlinearities followed by a max-pooling layer $L_{0}^{MP}$ with kernel 2×2 and stride 2. The second layer $L_{1}^{CV}$ convolves 128 two-dimensional filters with kernel 3×3 and stride 1, and again followed by a max-pooling layer $L_{1}^{MP}$ with kernel 2×2 and stride 2, The third layer $L_{2}^{CV}$ convolves 256 two-dimensional filters with kernel 3×3 and stride 1, and again followed by a max-pooling layer $L_{2}^{MP}$ with kernel 2×2 and stride 2. Then these feature maps are converted to a 512 dimensional vector by a fully-connected layer $L_{0}^{FC}$ with 512 units. The fully-connected layer $L^{FC}$ applies a linear transformation to the incoming data $x^{l-1}$:

$$x^{l}=\mathrm{ReLU}\left(W^{l}x^{l-1}+b^{l}\right).$$

The network also projects three frames of local goals and offset orientation angles, i.e., $\left(g_{t-2},g_{t-1},g_{t}\right)$ and $\left(\alpha_{t-2},\alpha_{t-1},\alpha_{t}\right)$, to a 9 dimensional vector. The network combines both vectors and feeds them to two fully-connected layers $L_{1}^{FC}$ and $L_{2}^{FC}$ with 512 units. Then the network applies a fully-connect layer $L_{3}^{FC}$ with 2 units without activations to produce the output, i.e., the mean of the action $a_{t}^{mean}=\left(v_{t}^{mean},\omega_{t}^{mean}\right)$, where $v_{t}^{mean}$ is the mean of the linear velocity and $\omega_{t}^{mean}$ is the mean of the angular velocity. The entire policy network $\pi_{\theta }$ can be expressed as follows:

$$a_{t}^{mean}=\prod_{k=1}^{3}L_{k}^{FC}\left(L_{0}^{FC}\left(\prod_{l=0}^{2}L_{l}^{MP}\cdot L_{l}^{CV}\left({\vec{M}}_{t}\right)\right)⊕{\vec{g}}_{t}⊕{\vec{\alpha }}_{t}\right),$$

where ⊕ is the concatenation operator. At last, the output of the network, i.e., the resulting action, is sampled from the Gaussian distribution $N(a_{t}^{mean},a_{t}^{logstd})$, where $a_{t}^{logstd}$ is a separate set of parameters referring to a log standard deviation, which will be updated during the training process. We also use a clip function to ensure that the resulting actions are valid in the action space.

The value network has the same architecture as the policy network, except the last layer is modified to only output the value $V_{\phi }\left({\vec{M}}_{t},{\vec{g}}_{t},{\vec{\alpha }}_{t}\right)$.

#### 3.2.3. Multi-Stage Curriculum Learning

Curriculum learning [54] aims to improve learning performance by designing appropriate curriculums for progressive learners from simple to difficult. Elman et al. [55] put forward the idea that a curriculum of progressively harder tasks could significantly accelerate a neural network’s training. Curriculum learning has recently become prevalent in the machine learning field, which assumes that the curriculum learning can improve the convergence speed of the training process and find a better local minimum value than the existing solvers. The formal expression of this idea can be found in [54]. The corresponding training distribution at step λ is

$$Q_{\lambda }\left(z\right)\propto W_{\lambda }\left(z\right)P\left(z\right)\;\;\forall z,$$

where *z* is a random variable representing an example for the learner, P(z) represents the target training distribution, $W_{\lambda }\left(z\right)\in \left[0,1\right]$ is the weight applied to example *z* at step $\lambda \in \left[0,1\right]$ in the curriculum sequence, and $W_{1}\left(z\right)=1$. The sequence of distributions $Q_{\lambda }$ is called a curriculum if the entropy of these distributions increases

$$H\left(Q_{\lambda }\right)<H\left(Q_{\lambda +\xi }\right)\;\;\forall \xi >0,$$

and $W_{\lambda }\left(z\right)$ is monotonically increasing in λ,

$$W_{\lambda +\xi }\left(z\right)⩾W_{\lambda }\left(z\right)\;\;\forall z,\forall \xi >0.$$

Increasing entropy means more diversity of training examples, and we want the weights of specific examples to increase as they are “added” to the training set. The curriculum strategy can be completed in two steps in the usual tasks: first a set of simple examples, and then the target training set.

**Figure 3:**
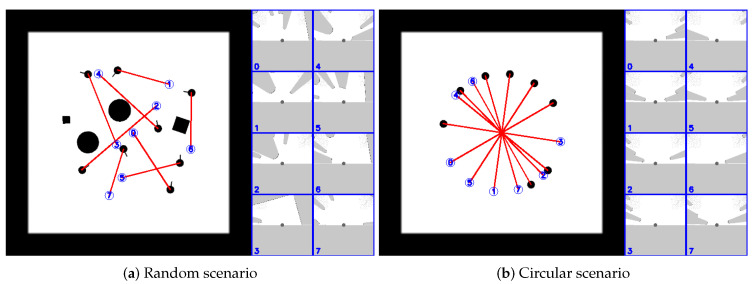
Two scenarios used to collect collision avoidance experiences for multiple robots, where the blue digital circles represent the target goals of the robot with the corresponding number, red lines specify the straight paths from the current position to the target for robots and other black pixels represent various obstacles. Blue boxes on the right illustrate egocentric local grid maps of each robot.

In this paper, we introduce a two-stage training process for curriculum learning. Specifically, we propose two scenarios, i.e., the random scenario and the circular scenario, that generate random environments in our customized simulator (as illustrated in Figure 3) to collect collision avoidance experiences for multiple robots. Figure 3a illustrates environments that randomly choose locations for obstacles, the starting and target positions of robots, which would help the robot to be able to avoid obstacles, a.k.a., random scenario. Environments in the random scenario are constructed in a 6×6 m${}^{2}$ space with eight robots and four obstacles, where the target position of each robot is randomly generated within the range of 2 m to 4 m from its starting point. Figure 3b illustrates environments that randomly place robots on a circle with a random radius, a.k.a., circular scenario, which helps the robot to be able to interact with other robots. Environments in the circular scenario randomly place eight robots on a circle with a varying radius in the range of 1.8 m to 3.0 m. These rich and complex training environments enable robots to explore their high-dimensional observation space and improve the quality and robustness of the learned policy. Combining with the centralized learning and the decentralized execution mechanism, the collision avoidance policy is effectively optimized at each iteration over these environments.

Following the idea of curriculum learning, we decompose our train process into two stages. In the first stage, we train the policy in environments of the random scenario (illustrated in Figure 3a) with eight robots and four obstacles. This allows robots to find a policy to avoid obstacles quickly. Once the policy has achieved an acceptable performance, we fix the trained policy and move to the second stage. In the second state, we continue to train the policy in environments of both the random scenario and circular scenario (illustrated in Figure 3b), where the number of robots is increased to 16.

**Figure 4:**
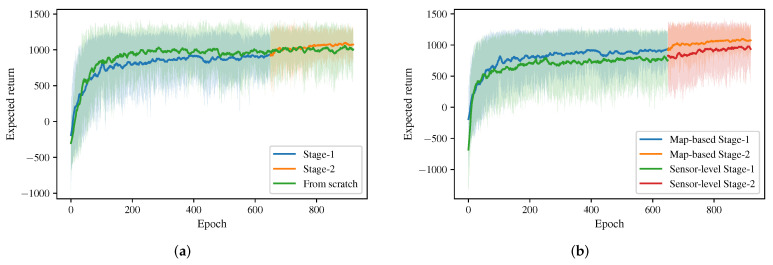
(**a**) Comparison of the expected return shown in epochs for the training process with two-stage curriculum learning and the training process without two-stage curriculum learning, i.e., from scratch. (**b**) Comparison of the expected return shown in epochs for the training process using Map-based policy and the training process using sensor-level policy.

Figure 4a shows the learning curve for an ablation study to illustrate the effect of two-stage curriculum learning, where “Stage-1” denotes the expected return for the training process in the first stage, “Stage-2” denotes the expected return for the training process in the second stage, and “From scratch” denotes the expected return for the training process without using two-stage curriculum learning. Note that two-stage curriculum learning helps the policy to converge with a higher expected return, i.e., a better collision avoidance performance.

## 4. Simulation Experiments

In this section, we evaluate our map-based multi-robot obstacle avoidance approach in various simulations environments. We first specify details of our implementation including the customized simulator, hyper-parameters, hardware and software for training the networks. Then, we quantitatively evaluate the performance of our map-based multi-robot navigation policy in various simulation scenarios and compare it with other existing approaches. For the specific performance of the robots in the experiments, please refer to the demonstration video at https://youtu.be/KOb1q23L7-U.

In the experiments, we implement three different approaches to generate corresponding multi-robot obstacle avoidance policies and compare them in multiple scenarios to evaluate the performance of these approaches. In specific, we consider the following policies:

- *NH-ORCA policy*: the policy generated by the state-of-the-art rule-based agent-level multi-robot collision avoidance approach proposed by Alonso-Mora et al. [18,19]. Hyper-parameters of the NH-ORCA algorithm used in our comparison experiments are listed in Table 1. For the details of definition of each parameter, please refer to [14].
- *Sensor-level policy*: the policy generated by the DRL based approach proposed by Long et al. [42] and Fan et al. [43]. Different from our approach, their network considers the original 2D laser data (in a 1D form) as the input and uses 1D convolutions to handle the input. For a fair comparison, we trained this DRL-based approach in the same training process, i.e., the two-stage training process, as our approach. The learning curve of its training process is shown in Figure 4b, where we denote their approach as “Sensor-level” and our approach as “Map-based”. Note that our approach converges with a higher expected return in the training process.
- *Map-based policy*: the policy generated by our approach in this article, which considers egocentric local grid maps as inputs.

To further evaluate the efforts of the two-stage curriculum learning in our approach, we specify the collision avoidance policy trained after the first training stage as “Map-based Stage-1” and the policy trained after the second stage as “Map-based Stage-2”.

We compare above collision avoidance policies from different perspectives, including the generalization to unseen scenarios, the efficiency for navigation and the robustness to the agent density and to the robots’ various shapes and dynamics. These experiments show that our map-based approach outperforms others in many indicators.

### 4.1. Reinforcement Learning Setup

We trained our Map-based agent following the DPPO algorithm in Algorithm 1 with the hyper-parameters listed in Table 2.

### 4.2. Implementation Details

Unlike our previous work [56] and most other related work that uses Gazebo [57] or Stage [58] as the simulator, our training environments are constructed by a customized simulator based on OpenCV (https://opencv.org/). In specific, the simulator can load a map of the environment as a gray image, where obstacles and robots are denoted as corresponding pixels in the image. Moreover, collisions can be identified as the coinciding of these pixels. Different from other simulators, like Gazebo, this customized simulator is efficient and flexible with lower communication delays.

We also implement differential drive robots in the simulator, whose pose (x,y,θ) is updated according to the velocity motion model of differential wheels:

$$\left(\begin{array}{c} x^{\prime }\\ y^{\prime }\\ \theta^{\prime } \end{array}\right)=\left(\begin{array}{c} x\\ y\\ \theta \end{array}\right)+\left(\begin{array}{c} -\frac{v}{\omega }\sin\theta +\frac{v}{\omega }\sin\left(\theta +\omega \Delta t\right)\\ \frac{v}{\omega }\cos\theta -\frac{v}{\omega }\cos\left(\theta +\omega \Delta t\right)\\ \omega \Delta t \end{array}\right),$$

where Δt is the interval time of velocity control, *v* and ω denote the linear velocity and the angular velocity, respectively. At each time step *t*, we intercept the world map according to the last pose of the robot *i* to generate the draft of the egocentric grid map ${\bar{M}}_{t}^{i}$. Then, we estimate the resulting egocentric local grid map $M_{t}^{i}$ from a laser scanner using the Bresenham’s line algorithm [59]. Note that we do not add any noise in environments constructed by the simulator in the training process, which helps us to optimize the policy with low variance.

Both the policy network and the value network are implemented in TensorFlow (https://www.tensorflow.org/) and trained with the Adam optimizer [52]. A computer with an i7-9900 CPU and an Nvidia Titan RTX GPU is used for the training. It takes around 12 hours to run about 900 iterations in Algorithm 1 to train the networks for converging in all the training scenarios. As specified in Table 2, the learning rate $lr_{\theta }$ of the policy network is set to $3\times 10^{-4}$ in the first training stage and is then reduced to $1\times 10^{-4}$ in the second training stage.

The learning policy can be executed online on robots in both the simulation and the real world in real-time. In the simulation, it takes 2 ms on the GPU for the policy network to compute new actions for 8 robots. After deploying the policy to robots in the real work, it takes about 25 ms on an Nvidia Jetson TX2 for policy network to compute new actions and takes about 30 ms to generate egocentric local grid maps from the 2D laser scanning data.

### 4.3. Generalization Capability

Notice that we only use environments from the random scenario and the circular scenario to train the networks for multi-robot collision avoidance in our map-based DPPO approach. We demonstrate the generalization of our approach by showing that the learned policy performs well to a series of unseen scenarios and also performs well for heterogeneous robots after introducing robots with different sizes and shapes in the training.

#### 4.3.1. Non-Cooperative Robots

**Figure 5:**
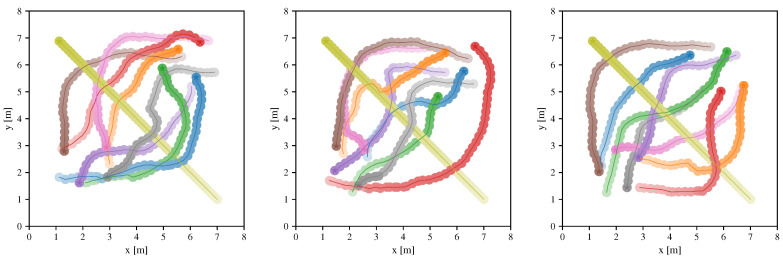
The scenario with non-cooperative robots, where eight robots are controlled by the learned policy to interact with a non-cooperative robot. The non-cooperative robot (with the yellow color) moves along a straight line at a speed of 0.5 m/s, robots represented by other colors are controlled by the same learned collision avoidance policy.

Here we introduce a scenario that contains a non-cooperative robot, i.e., a moving robot that can not be affected by other robots. Figure 5 illustrates three environments of the scenario, where eight robots are deployed with the collision avoidance policy generated by our approach, and the robot with the yellow color moves along a straight line at a speed of 0.5 m/s.

The experimental results show that, though non-cooperative robots are not introduced in the training process, the learned policy can still allow other robots to avoid the collision with it.

#### 4.3.2. Large-Scale Scenarios

**Figure 6:**
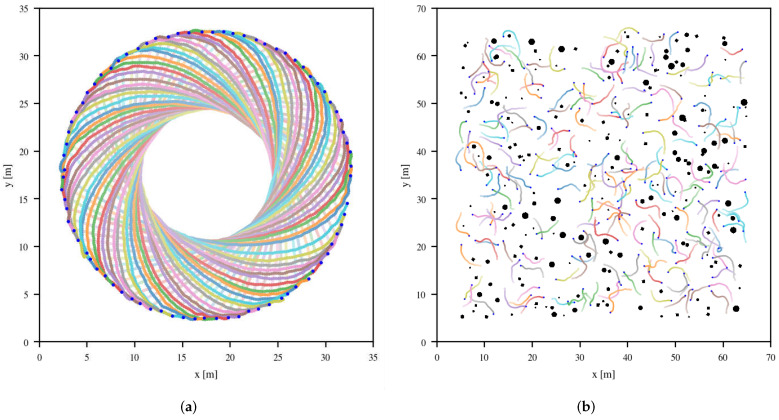
(**a**) The large-scale scenario extends from the circular scenario, which uniformly places 80 robots on a large circle and asks them to move to their antipodal positions. The blue points denote robots’ target positions. (**b**) The large-scale scenario extends from the random scenario, which randomly places 200 robots and 200 obstacles with different sizes in an area with 60×60 $m^{2}$ and asks them to move to random-choosing target positions within the range of 4.5 m to 5 m relative to corresponding starting positions. The blue points also denote robots’ target positions.

We also introduce two large-scale scenarios to demonstrate the generalization of the learned policy. In specific, the first large-scale scenario extends from the circular scenario that uniformly places 80 robots on a large circle and asks them to move to their antipodal positions. An environment of the scenario is illustrated in Figure 6a. The second large-scale scenario extends from the random scenario that randomly places 200 robots and 200 obstacles with different sizes in an area with 60×60 $m^{2}$ and asks them to move to random-choosing target positions within the range of 4.5 m to 5 m relative to corresponding starting positions. An environment of the scenario is illustrated in Figure 6b.

The experimental results show that, though both large-scale scenarios are unseen for the learned policy, it can still perform well in environments of both scenarios without fine-tuning.

#### 4.3.3. Heterogeneous Robots

**Figure 7:**
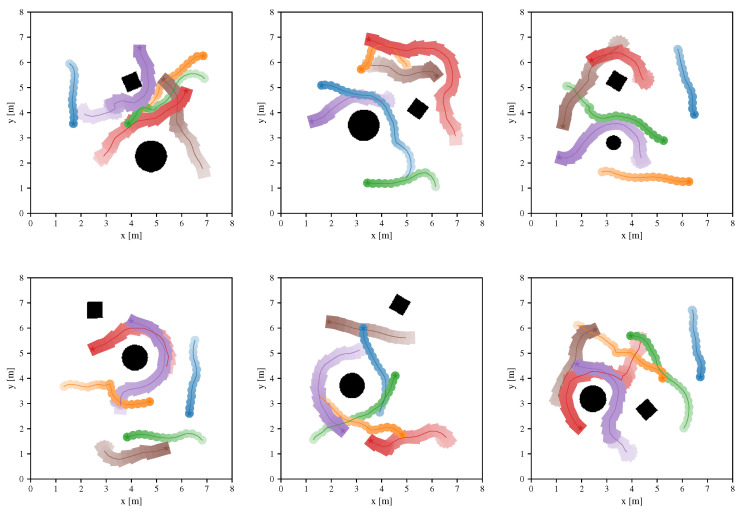
The scenario with heterogeneous robots, which contains three rectangular robots, three circular robots and obstacles with different shapes. All robots are controlled by the same learned collision avoidance policy.

Now we introduce a scenario with heterogeneous robots, i.e., robots with different sizes and shapes. Figure 7 illustrates six environments of the scenario, which contains three rectangular robots, three circular robots and obstacles with different shapes.

The experimental results show that, after introducing corresponding robots with different shapes in the training, the learned policy can also perform well in environments of this scenario.

### 4.4. Efficiency Evaluation

In this section, we evaluate the efficiency of navigation of the learned collision avoidance policy. We first introduce some metrics to evaluate the performance of approaches for the term. Then we compare different policies using these metrics.

#### 4.4.1. Metrics and Scenarios

We introduce four metrics to evaluate the efficiency of navigation of the policies w.r.t. different approaches as the following:

- *Success rate $\bar{\pi }$*: the ratio of the episodes that end with the robot reaching its target without any collision.
- *Extra time $\bar{t}$*: the average time required for every robot to successfully reach their targets without any collisions minus the average time for every robot to drive straight to their targets with the maximum speed.
- *Extra distance $\bar{d}$*: the average moving distance required for every robot to successfully reach their targets without any collisions minus the average moving distance for every robot to drive straight to their targets with the maximum speed.
- *Average linear velocity $\bar{v}$*: the average linear velocity for every robot during the navigation.

We will compare the efficiency for navigation of four policies, i.e., NH-ORCA policy, Sensor-level policy, Map-based Stage-1 policy and Map-based Stage-2 policy. All of these metrics with be calculated from the averaging results of 100 different environments of the testing scenarios in each case.

Now we introduce the testing scenarios used in this paper.

- *Circle scenario*: the scenario that is similar to the circular scenario, except that the initial positions of all robots are uniformly placed on the circle. This scenario can be categorized into four types by the different number of robots and the radius of the circle, i.e., 6 robots with radius 2.5 m, 8 robots with radius 3 m, 10 robots with radius 3.5 m and 12 robots with radius 3.5 m.
- *Cross scenario*: the scenario that requires two groups of robots (four robots in each group) to move cross each other.
- *Swap scenario*: the scenario that requires two groups of robots (four robots in each group) to move towards each other and swap their positions.
- *New random scenario*: the scenario that is similar to the random scenario, except that 10 robots are considered in the scenario.

#### 4.4.2. Quantitative Results

**Figure 8:**
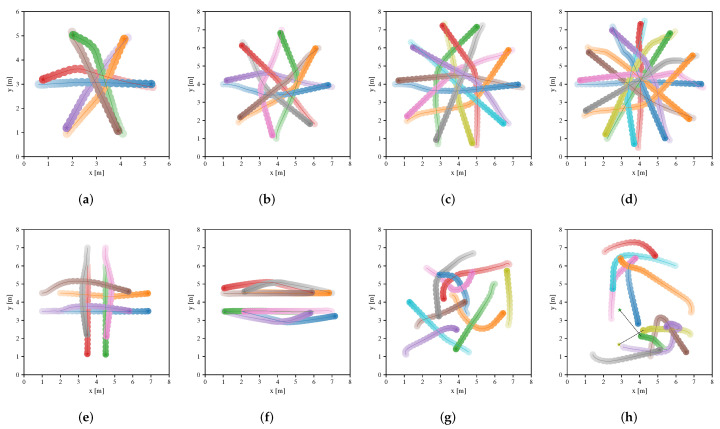
Trajectories of robots executing NH-ORCA policy in environments of the circle scenario (**a**–**d**), cross scenario (**e**), swap scenario (**f**) and new random scenario (**g**,**h**), respectively. The trajectories of different robots are distinguished by different colors, and color transparency is used to indicate the time state along each trajectory.

**Figure 9:**
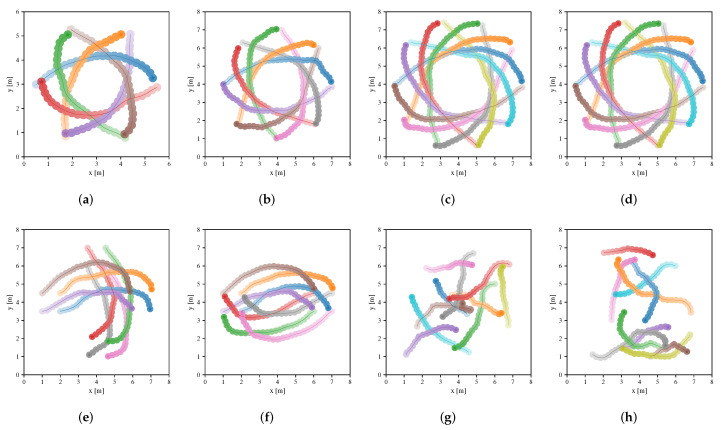
Trajectories of robots executing Sensor-level policy in environments of the circle scenario (**a**–**d**), cross scenario (**e**), swap scenario (**f**) and new random scenario (**g**,**h**), respectively. The trajectories of different robots are distinguished by different colors, and color transparency is used to indicate the time state along each trajectory.

**Figure 10:**
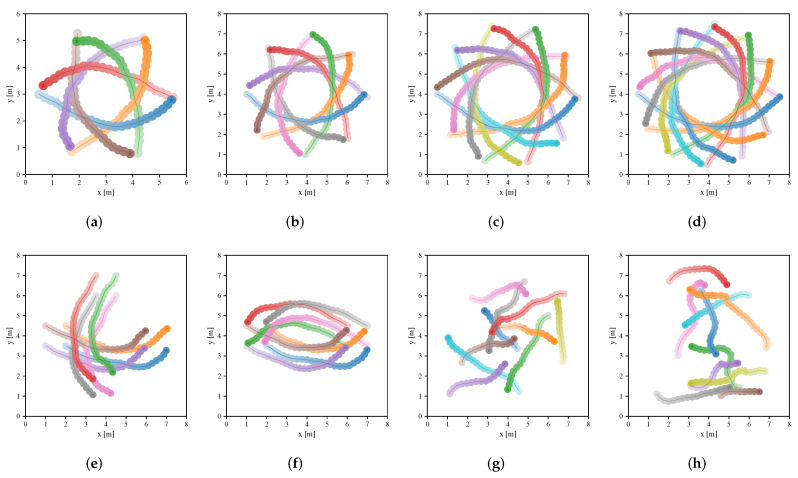
Trajectories of robots executing Map-based Stage-2 policy in environments of the circle scenario (**a**–**d**), cross scenario (**e**), swap scenario (**f**) and new random scenario (**g**,**h**), respectively. The trajectories of different robots are distinguished by different colors, and color transparency is used to indicate the time state along each trajectory.

Table 3 summarizes the performance metrics of four approaches in the testing scenarios, where metrics are calculated from the averaging results of 100 different environments of the scenarios in each case. Note that Map-based Stage-2 policy outperforms others in terms of extra time and average speed. The success rate of both Map-based Stage-2 policy and Sensor-level policy are both 1.0 in many scenarios. However, Sensor-level policy does not perform well in other metrics compared to Map-based Stage-2 policy, which indicates that the policy learned by the sensor-level approach can be further optimized by the map-based approach. NH-ORCA policy is a non-cooperative policy, where each robot performs greedily resulting in a small extra distance (as illustrated in Figure 8), but the poor performance of other metrics. For Map-based Stage-1 policy, we can see that it has a good performance in migrating to the circle scenario, which indicates that the policy can be generalized to unseen scenarios since the circular scenario is not considered in the first training stage. Note that the performance of the policy is greatly improved, after training in the second stage. We also illustrate the performance of Sensor-level policy and Map-based Stage-2 policy in the testing scenarios in Figure 9 and Figure 10, respectively.

### 4.5. Robustness Evaluation

In addition to the generalization and the navigation efficiency, the learned policy is also preferred to be stable and robust to model uncertainty and input noises. We are to verify the robustness of our learned policy by multiple experiments in this section.

#### 4.5.1. Different Sensor Noise

**Figure 11:**
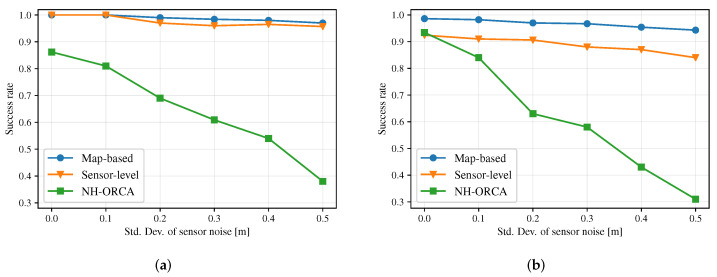
(**a**) Comparison of the success rate of NH-ORCA policy, Sensor-level policy and Map-based policy in the circle scenario with 12 robots, when the standard deviation of sensor noise increases. (**b**) Comparison of success rates between the Map-based policy, Sensor-level policy and NH-ORCA in the new random scenario with 10 robots, when the standard deviation of sensor noise increases.

Sensor noise and perceptual errors are common in robot systems. To verify the ability of different approaches to resist noise, we gradually increase the error of laser sensing distance data for Sensor-level policy and our Map-based (Stage-2) policy, and gradually increase the perception error of surrounding robots’ positions and velocities for NH-ORCA policy. Figure 11 depicts that the success rate of all three policies in both the circle scenario with 12 robots and the new random scenario with 10 robots when the variance of Gaussian noise increases. The results show that DRL-based policies, i.e., Map-based and Sensor-level polices, are resilient to noise, which sensor noise affects the success rate of NH-ORCA policy greatly.

#### 4.5.2. Different FOV Limits

**Figure 12:**
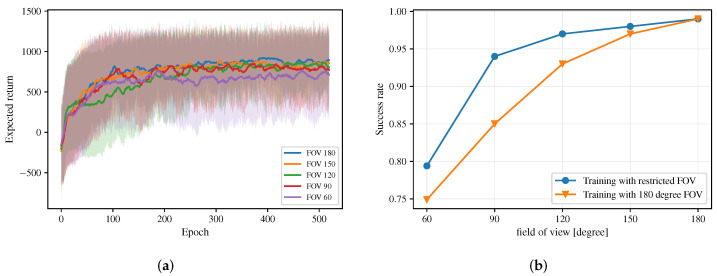
(**a**) Comparison of expected returns of the trained Map-based policies for sensors with different Fields of View (FOVs). (**b**) Comparison of the success rate between the trained Map-based policy with specific restricted FOVs and that with 180-degree FOV. training with specific restricted FOV and the Map-based policy training with 180-degree FOV in the new random scenario.

Different sensors have different FOVs, which also affects the performance of the collision avoidance policy greatly. We consider five laser sensors with different FOVs and apply our Map-based DPPO approach on their sensor data to train corresponding Map-based policies. Figure 12a shows the learning curves of these Map-based policies and Figure 12b shows the success rate of these policies in the new random scenario. Moreover, we also test the Map-based policy trained by the sensor with 180-degree FOV on robots with restricted FOVs. The result shows that the policy performs better when robots have the same FOV in both training and testing scenarios.

#### 4.5.3. Different Sensor Types

**Figure 13:**
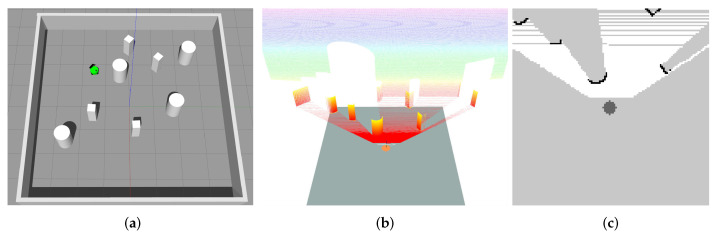
(**a**) An environment contains a robot (green) with a depth camera and randomly placed obstacles (white) in Gazebo. (**b**) Point cloud data generated by the depth camera with 120-degree FOV. (**c**) The grid map generated from the point cloud data.

**Figure 14:**
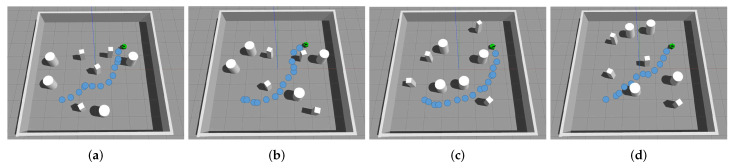
The schematic diagram of the trajectory of the robot generated by the learning Map-based policy using the depth camera in four simulation environments (**a**–**d**) with 8 randomly placed obstacles.

As discussed in Section 1, the map-based approach can be easily deployed to robots with different sensors. Here we illustrate such an example by applying the approach to a robot with only a depth camera. Since our customized simulator lacks 3D information, we choose Gazebo, a more realistic 3D simulator, to test the generalization ability of our model. In specific, we implement a differential robot with a depth camera with a 120-degree horizontal FOV and 640 × 480 resolution in Gazebo. We are to convert the point cloud data generated by the depth camera into corresponding grid maps. We first down-sample the point cloud data to the resolution of the map, remove outliers, refine the result by eliminating the ceiling, then generate the grid map from the bird’s eye view of the resulting point cloud. Figure 13 illustrates the procedure of how to generate such a grid map from the point cloud data. An example of the Using the generated grid map, we can construct the corresponding egocentric local grid map, which can later be used to train the collision avoidance policy by our map-based approach. Figure 14 shows the performance of Map-based policy using the depth camera in environments with 8 randomly placed obstacles. Note that the robot successfully avoids all obstacles and reaches its target the depth camera only.

## 5. Real-World Experiments

In this section, we deploy the trained Map-based policy to real robots to perform multi-robot obstacle avoidance in the real world. As discussed in Section 1, the map-based approach is more robust to noisy sensor data, does not require robots’ movement data and considers sizes and shapes of related robots, which make it more efficient and easier to be deployed to real robots. However, there are more challenges in deploying the learned collision avoidance policy from simulation to the real world. As discussed in [43], besides noisy sensor data, the clocks of each individual robot are not synchronized with each other, which results in an asynchronous distributed system hard to control, and robots cannot provide consistent behavior with the same control command, due to many realistic factors such as mechanical details, motor characteristics and the approximation with friction model. In addition to the generalization capabilities and robustness on simulation environments as discussed in Section 4, the real-world experiments show that the map-based approach can be easily deployed to real robots and performs well in the real world. For the specific performance of the robots in the experiments, please refer to the demonstration video at https://youtu.be/KOb1q23L7-U.

In the following, we first introduce the hardware setup of real robots. Then, we evaluate the performance of these robots in multiple scenarios in the real world. At last, we demonstrate long-range navigation of the robot with our learned map-based collision avoidance policy in a corridor environment.

### 5.1. Hardware Setup

**Figure 15:**
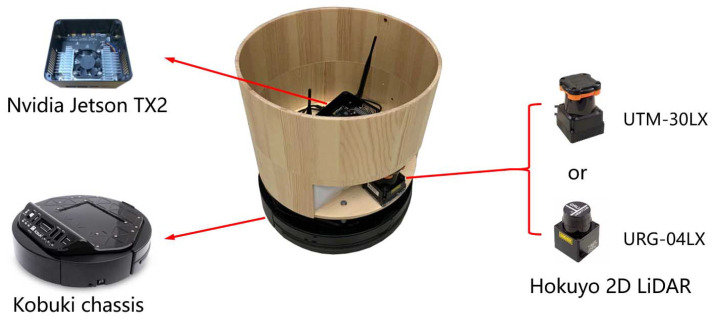
The robot is based on TurtleBot 2 with Kobuki base using either a Hokuyo UTM-30LX 2D LiDAR or a Hokuyo URG-04LX 2D LiDAR and an NVIDIA Jetson TX2. A wooden shell is placed on the top of the mobile platform to enable robots to detect each other.

We deploy the Map-based policy to four robots to perform multi-robot obstacle avoidance in the real world. As shown in Figure 15, each robot is based on TurtleBot 2 with Kobuki base. Then the robot has a circular shape with a radius of 0.17 m. The laser sensor used in each robot is either a Hokuyo UTM-30LX 2D LiDAR or a Hokuyo URG-04LX 2D LiDAR (which has a lower price). The details of the Hokuyo 2D LiDAR sensors are listed in Table 4. All of these sensors provide a 180-degree FOV observation of the surrounding environment in front of the robot. The robot applies an NVIDIA Jetson TX2 as its computing platform. In specific, we implemented four versions of such a robot, where two of them are equipped with Hokuyo UTM-30LX 2D LiDARs and two of them are Hokuyo URG-04LX 2D LiDARs.

In experiments, the relative local goals of the robot are provided by a particle filter based on a state estimator. The egocentric local grid map is constructed from the laser data, which has a fixed size 6.0 × 6.0 m and the resolution 0.1 m at each time step. A wooden shell is placed on the top of the mobile platform of the robot, which enables robots to detect each other. We also use paper boxes and luggages to act as static and dynamic obstacles in tests. The output of the trained policy network is directly used to control the robot, that is, the range of robot linear velocity and angular velocity are $v_{t}\in \left[0,\;0.6\right]$ (in meters per second) and $\omega_{t}\in \left[-0.9,\;0.9\right]$ (in radians per second), respectively.

### 5.2. Static and Dynamic Scenarios

In this section, we introduce a series of real-world scenarios for a single robot to evaluate the performance of the collision avoidance policy generated by our map-based DPPO approach.

**Figure 16:**
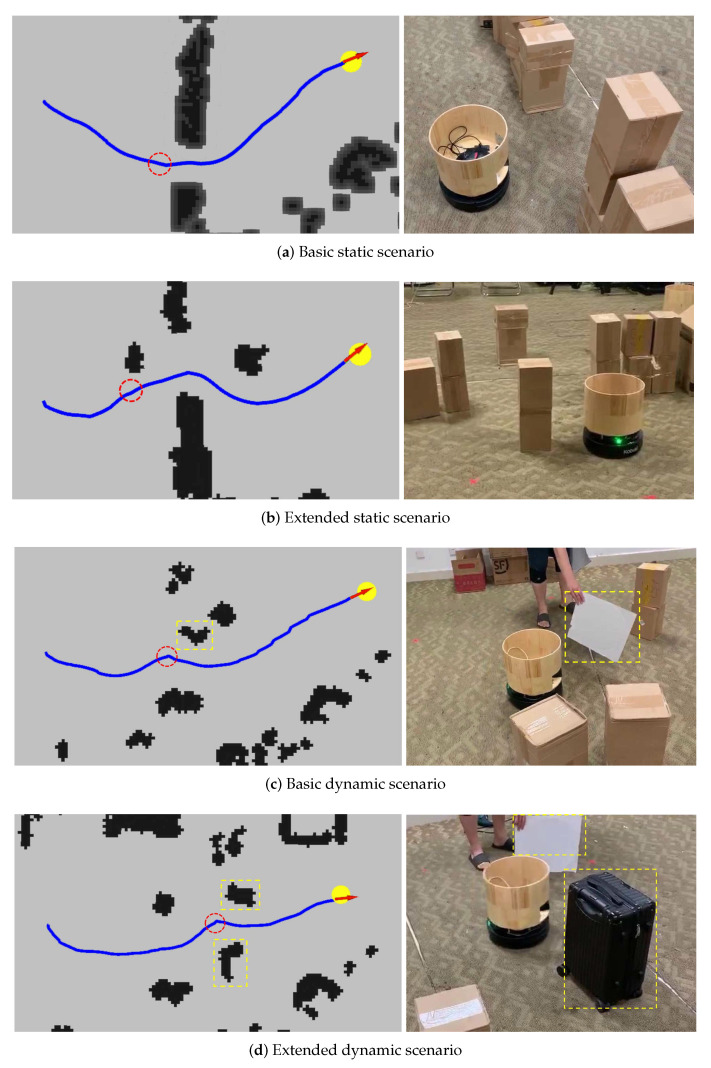
(**a**) The basic static scenario, where some paper boxes are places as static obstacles to block the robot’s path from its starting position to its target. (**b**) The extended static scenario, where two obstacles are added to the front and the rear of the narrow opening of a line of obstacles. (**c**) The basic dynamic scenario, where cardboard appears suddenly in the moving path of the robot. (**d**) The extended dynamic scenario, where cardboard appears suddenly and a luggage moves across the path of the robot. Subfigures on the left show the trajectories and the grid maps of the robot. Subfigures on the right show the environments in the real world, which correspond to situations that the robot is at the place of the red circle in corresponding left figures. Yellow bounding boxes in the figures denote corresponding dynamic obstacles in the environments.

We first introduce a basic static scenario where some paper boxes are places as static obstacles to block the robot’s path from its starting position to its target. Figure 16a illustrates an environment of this scenario, in which the robot can successfully pass through a line of obstacles with a narrow opening. Next, we extend the scenario by adding obstacles to the front and the rear of the opening to increase the difficulty. Figure 16b illustrates an environment of such scenario, in which the robot can still successfully pass through the narrow opening. Then we introduce dynamic scenarios by allowing some obstacles to appear suddenly. In specific, Figure 16c illustrates an environment in which a cardboard appears suddenly in the moving path of the robot. Figure 16d illustrates an environment extended from the situation in Figure 16c, where a luggage moves across the path of the robot. In both environments, the robot successfully detects the dynamic obstacles and adjusts its movement in time to avoid the collision.

More demonstrations on these static and dynamic scenarios can be found in the demonstration video. These experiments show that the robot using our learned collision avoidance policy performs well in these scenarios.

### 5.3. Multi-Robot Scenarios

In this section, we introduce a serious of real-world scenarios for multiple robots to evaluate the performance of the multi-robot collision avoidance policy generated by our map-based DPPO approach. Moreover, both static obstacles and dynamic pedestrians are also added in these scenarios.

**Figure 17:**
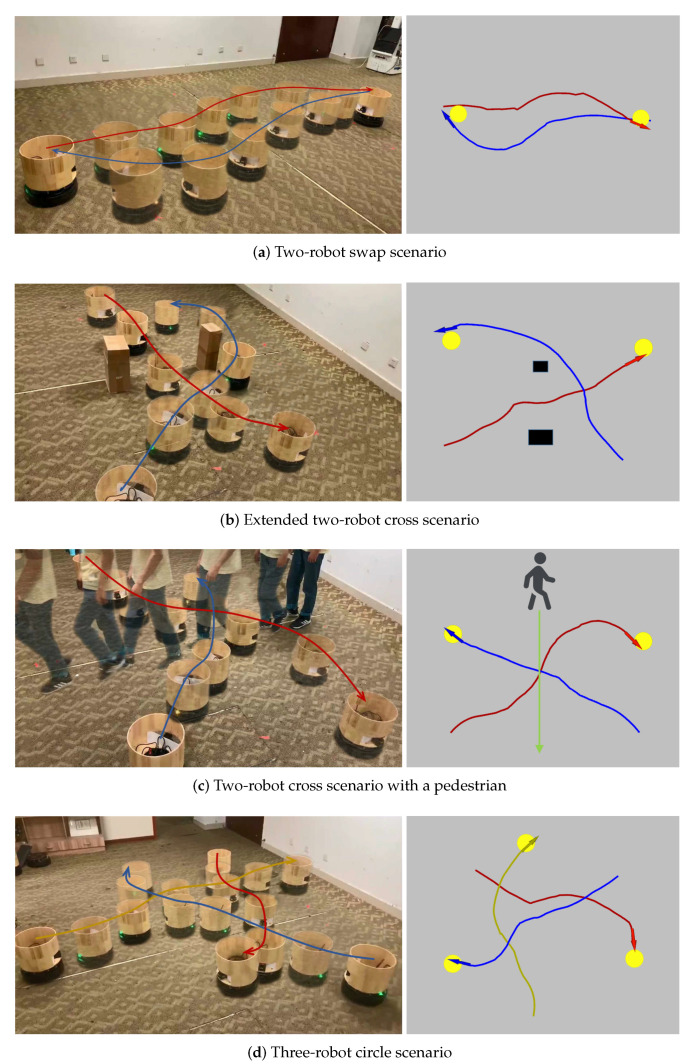
(**a**) Two-robot swap scenario, where two robots are required to move towards each other and swap their positions. (**b**) Extended two-robot cross scenario, which is extended from the two-robot cross scenario by randomly placing some static obstacles. (**c**) Two-robot cross scenario with a pedestrian, where a pedestrian is asked to walk across the paths of both robots. (**d**) Three-robot circle scenario, where three robots are placed on a circle and their targets are set at opposite positions. Subfigures on the left show the trajectories of the robots in the real world. Subfigures on the right show the trajectories recorded by robots based on their own localizations. Notice that the trajectories recorded by robots are not that smooth which is mainly due to the low accuracy of the robots’ localizations. Lines with different colors denote trajectories of different robots.

**Figure 18:**
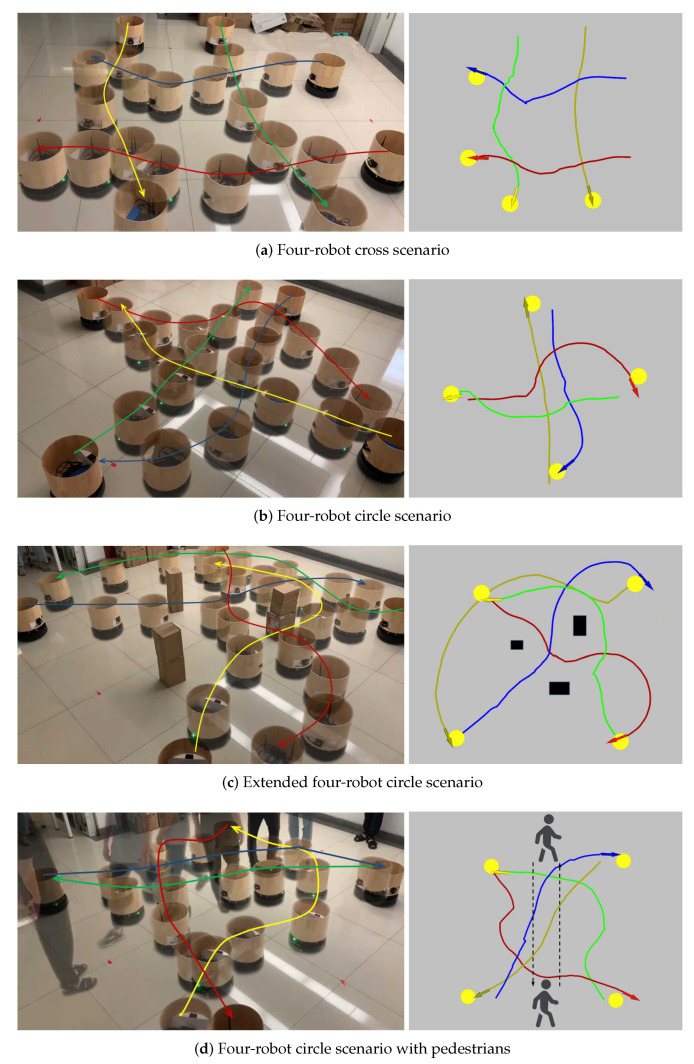
(**a**) Four-robot cross scenario, where two groups of robots cross each other. (**b**) Four-robot circle scenario, where four robots are placed on a circle and their targets are set at opposite positions. (**c**) Extended four-robot circle scenario, which is extended from the four-robot circle scenario by randomly placing some static obstacles. (**d**) Four-robot circle scenario with pedestrians, where two pedestrians are asked to walk across the paths of all robots. Subfigures on the left show the trajectories of the robots in the real world. Subfigures on the right show the trajectories recorded by robots based on their own localizations. Notice that the trajectories recorded by robots are not that smooth which is mainly due to the low accuracy of the robots’ localizations. Lines with different colors denote trajectories of different robots.

We first introduce a real-world swap scenario where two robots are required to move towards each other and swap their positions. Figure 17a illustrates the performance of the robots in this scenario, in which both robots successfully reach their targets without collision. Next, we extend the two-robot cross scenario by randomly placing some static obstacles to increase the difficulty. Figure 17b illustrates the performance of the robots in the new scenario, in which both robots still successfully reach their targets without collision. Figure 17b also shows that the robot can automatically wait for a moment and avoid the narrow opening when it observes that the other robot is going through the opening. Then, we further extend the scenario by asking a pedestrian to walk across the paths of both robots. Figure 17c illustrates the performance of the robots in this extended scenario, in which both robots adaptively slow down their speed and wait for the pass of the pedestrian before they each their targets successfully. Notice that pedestrians are not introduced in the training procedure of the collision avoidance policy in our approach and the shape and dynamic characteristics of pedestrians are quite different from other robots and static obstacles. However, the learned policy still performs well in the scenario with pedestrians. Later, we extend the two-robot scenario to involve three robots. We introduce the three-robot circle scenario where three robots are placed on a circle and their targets are set at opposite positions on the circle. Figure 17d illustrates the performance of the robots in this scenario, in which all robots successfully reach their targets without collision. At last, we extend these scenarios to involve four robots. Figure 18 illustrates the performances of four robots in these scenarios, in which all robots still successfully reach their targets without collision. In the four-robot cross scenario shown in Figure 18a, two groups of robots cross each other and reach their respective target points. Figure 18b shows the experimental results of the four-robot circle scenario, where four robots are placed on a circle and their targets are set at opposite positions. In order to further prove the generalization ability of our model in more complex environments, we added three static obstacles or two pedestrians to the four-robot circle scenario. The experimental results are shown in Figure 18c,d. All robots can reach their respective target points without collision in such a dynamic and crowded environment.

More demonstrations on these multi-robot scenarios can be found in the demonstration video. These experiments show that, though the dynamic characteristics of both robots in the real world are different from the simulation robots in the simulator, our learned map-based collision avoidance policy can still perform well in these scenarios.

### 5.4. Long-Range Navigation

Note that the learned collision avoidance policy is used to consist of the navigation system for robots. We further verify the effectiveness of the entire navigation system with our DRL-based collision avoidance policy for long-range navigation.

In specific, we present a hierarchical architecture [56] for long-range navigation that combines A* path planning [60] with the DRL-based collision avoidance policy generated by our map-based DPPO approach, where the short-range DRL-based motion planner directly maps egocentric local grid maps to robot’s control commands in terms of intermediate goals provided by the global A* path planner. In the navigation procedure, each local goal position is selected as a position that is 3 m away from the robot on the global planned path.

**Figure 19:**
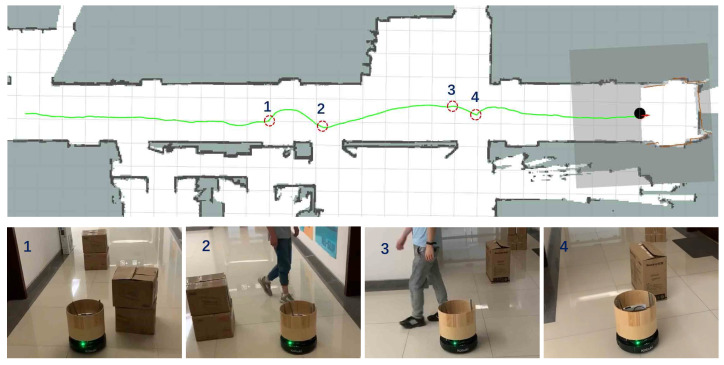
The long-range navigation scenario, where the navigation system with our DRL-based collision avoidance policy to go through the long corridor while avoiding static obstacles and dynamic pedestrians. The upper picture denotes the grid map of the entire environment map and the trajectory of the robot, and the square around the robot represents its egocentric local grip map. The lower pictures specify situations of the corresponding number in the real world when the robot is at the corresponding dotted circles in the map.

This navigation system with the DRL-based collision avoidance policy is evaluated in environments with long corridors (about 27 m). We use the open source SLAM algorithm Cartographer [61] to build a global static map of the environment in advance. The global static map is used for particle filter-based positioning and path planning, which provide our DRL-based collision avoidance module with the pose of the local target point relative to the robot. Figure 19 illustrates such an environment with its grid map and the trajectory generated by the navigation system, where the robot can successfully go through the long corridor while avoiding static obstacles and dynamic pedestrians.

## 6. Conclusions

In this paper, we propose a map-based DPPO approach for multi-robot collision avoidance in distributed and communication-free environments. We use the egocentric local grid map of a robot to represent the environmental information around it including its shape and observable appearances of other robots and obstacles, which can be easily generated by using multiple sensors or sensor fusion. Then we apply DPPO to train a convolutional neural network that directly maps three frames of egocentric local grid maps and the robot’s relative local goal positions into low-level robot control commands. We apply a two stage training procedure to train networks using environments from the random scenario and the circular scenario.

We evaluate the learned collision avoidance policy in multiple simulation scenarios and compare it with related work. The experimental results show the outstanding performance of our approach in terms of success rate, extra time as well as average linear velocity. We also evaluate our learned policy from different perspectives, including the generalization to unseen scenarios, the efficiency for navigation and the robustness to the agent density and to the robots’ various shapes and dynamics. These experiments show that our map-based approach outperforms others in many indicators. Then, we deploy the trained model to real robots to evaluate its performance in various real-world scenarios, including environments with static and dynamic obstacles, multiple robots and dynamic pedestrians. These experiments show that our approach is efficient and easy to deploy to real robots, and performs well in the real world. At last, we integrate our DRL-based collision avoidance policy into the navigation framework and test it in long-range navigation. The experiment shows that the navigation system can successfully lead the robot to go through a long corridor while avoiding static obstacles and dynamic pedestrians.

Our future work intends to use other multi-sensor fusion solutions to take advantage of our Map-based method, and utilize other positioning methods (such as UWB technology or visual SLAM) to solve the error-prone problem of laser positioning in dense environments, and finally deploy our map-based DRL navigation to more dynamic and crowding environment, such as canteens and vegetable markets.

## Figures and Tables

**Table 1:** Hyper-parameters of the NH-ORCA algorithm used in our comparison experiments.

Parameter	Value
VO type	HRVO
Use ORCA	True
Use clearpath	True
Epsilon	0.1
Time horizon	10
Time to holonomic	0.4
Minimum tracking error	0.02
Maximum tracking error	0.1

**Table 2:** Hyper-parameters of our training algorithm described in Algorithm  1.

Parameter	Value
$T_{ep}$ in line 5	2000
$T_{m}$ in line 12	200
$d_{arr}$ in line 14	0.2
λ in line 19	0.95
γ in line 19 and 35	0.99
$E_{\pi }$ in line 26	80
ε in line 27	0.2
$KL_{target}$ in line 28	0.01
$lr_{\theta }$ in line 31	$3.0\times 10^{-4}$ (Stage 1), $1.0\times 10^{-4}$ (Stage 2)
$E_{v}$ in line 34	80
$lr_{\phi }$ in line 36	$1.0\times 10^{-3}$

**Table 3:** Performance metrics (as mean/standard deviation) evaluated for different methods on different scenarios with varied scene sizes and a different number of robots.

Scenarios (Agents, Range)	Method	$\bar{\pi }$	$\bar{t}$ (mean/std)	$\bar{d}$ (mean/std)	$\bar{v}$ (mean/std)
Circle scenario (6, radius 2.5 m)	NH-ORCA	0.969	2.6676/1.3981	**0.2004/0.1160**	0.4490/0.1537
	Sensor-level	**1.000**	2.0620/0.5576	0.8773/0.2269	0.5636/0.1328
	Map-based Stage-1	0.937	8.2528/6.4266	0.7861/0.4763	0.3328/0.2881
	Map-based Stage-2	**1.000**	**2.0000/0.3502**	0.8648/0.1447	**0.5659/0.1283**
Circle scenario (8, radius 3 m)	NH-ORCA	0.950	3.4988/1.9744	**0.2057/0.1299**	0.4479/0.1520
	Sensor-level	**1.000**	2.5400/0.5084	1.1992/0.1918	0.5687/0.1233
	Map-based Stage-1	0.914	10.3488/6.3236	0.9185/0.5446	0.3218/0.2880
	Map-based Stage-2	**1.000**	**2.3170/0.2577**	1.0204/0.1513	**0.5730/0.1146**
Circle scenario (10, radius 3.5 m)	NH-ORCA	0.892	4.2930/2.6132	**0.2486/0.1983**	0.4366/0.1546
	Sensor-level	**1.000**	3.3045/0.4784	1.5991/0.2145	0.5734/0.1142
	Map-based Stage-1	0.903	11.9304/9.2772	1.0635/0.6968	0.3212/0.2867
	Map-based Stage-2	**1.000**	**2.5881/0.4650**	1.1870/0.1710	**0.5735/0.1114**
Circle scenario (12, radius 3.5 m)	NH-ORCA	0.862	5.2137/3.4742	**0.2817/0.2599**	0.4078/0.1711
	Sensor-level	**1.000**	3.7290/0.5355	1.7884/0.2525	0.5699/0.1170
	Map-based Stage-1	0.873	15.7697/11.7475	1.0773/0.7475	0.2698/0.2871
	Map-based Stage-2	**1.000**	**2.6133/0.4527**	1.2170/0.1769	**0.5745/0.1120**
Cross scenario (8, 8×8 m${}^{2}$)	NH-ORCA	0.958	2.1283/1.5166	**0.1883/0.2081**	0.4851/0.1430
	Sensor-level	0.995	2.8238/1.2894	1.1174/0.5214	0.5419/0.1588
	Map-based Stage-1	0.950	4.0802/3.4952	1.0158/0.7322	0.4764/0.2278
	Map-based Stage-2	**1.000**	**1.8315/1.2333**	0.7873/0.4912	**0.5608/0.1384**
Swap scenario (8, 8×8 m${}^{2}$)	NH-ORCA	0.906	2.2174/2.1307	**0.2651/0.2228**	0.4845/0.1648
	Sensor-level	**1.000**	2.7357/0.9494	1.1498/0.3479	0.5535/0.1419
	Map-based Stage-1	0.994	2.7272/2.2479	0.8017/0.5761	0.5206/0.1874
	Map-based Stage-2	**1.000**	**2.0201/1.0430**	0.9816/0.3660	**0.5584/0.1424**
New random scenario (10, 8×8 m${}^{2}$)	NH-ORCA	0.934	4.3181/3.1353	0.5697/0.6412	0.3760/0.1890
	Sensor-level	0.924	3.4519/3.4162	0.5417/0.5048	0.4017/0.2687
	Map-based Stage-1	0.955	3.1650/2.5632	0.5514/0.4643	0.4202/0.2590
	Map-based Stage-2	**0.986**	**2.9009/2.4523**	**0.4531/0.3610**	**0.4460/0.2497**

**Table 4:** The details of the Hokuyo 2D LiDAR sensors.

Model No.	UTM-30LX	URG-04LX
Measuring area	0.1 to 30 m, $270^{\circ }$	0.02 to 5.6 m, $240^{\circ }$
Accuracy	0.1 to 10 m: ±30 mm, 10 to 30 m: ±50 mm	0.06 to 1 m: ±30 mm, 1 to 4.095 m: ±3%
Angular resolution	$0.25^{\circ }$	$0.36^{\circ }$
Scanning time	25 ms/scan	100 ms/scan
